# Curvature Induced Modifications of Chirality and Magnetic Configuration in Perpendicular Films

**DOI:** 10.1021/acsnano.5c08926

**Published:** 2025-08-11

**Authors:** David Raftrey, Dhritiman Bhattacharya, Colin Langton, Bradley J. Fugetta, Subhashree Satapathy, Olha Bezsmertna, Andrea Sorrentino, Denys Makarov, Gen Yin, Peter Fischer, Kai Liu

**Affiliations:** † Materials Sciences Division, 1666 Lawrence Berkeley National Laboratory, Berkeley, California 94720, United States; ‡ Department of Physics, University of California Santa Cruz, Santa Cruz, California 95064, United States; § Department of Physics, 8368 Georgetown University, Washington, District of Columbia 20057, United States; ∥ Institute of Ion Beam Physics and Materials Research, 28414 Helmholtz-Zentrum Dresden-Rossendorf e.V., 01328 Dresden, Germany; ⊥ Alba Light Source, MISTRAL Beamline, 08290 Cerdanyola del Vallès, Spain

**Keywords:** 3D nanomagnetism, noncollinear spin textures, magnetic X-ray nanotomography, curvature effects, nanowires, Dzyaloshinskii–Moriya interaction

## Abstract

Designing curvature in three-dimensional (3D) magnetic nanostructures enables controlled manipulation of local energy landscapes, allowing for the modification of noncollinear spin textures relevant for next-generation spintronic devices. In this study, we experimentally investigate 3D magnetization textures in a Co/Pd multilayer film, exhibiting strong perpendicular magnetic anisotropy (PMA), deposited onto curved Cu nanowire meshes with diameters as small as 50 nm and lengths of several microns. Utilizing magnetic soft X-ray nanotomography, we achieve reconstructions of 3D magnetic domain patterns at approximately 30 nm spatial resolution. This approach provides detailed information on both the orientation and magnitude of magnetization within the film. Our results reveal that interfacial anisotropy in the Co/Pd multilayers drives the magnetization toward the local surface normal. In contrast to typical labyrinth domains observed in planar films, the presence of curved nanowires significantly alters the domain structure, with domains preferentially aligning along the nanowire axis in close proximity, while adopting random orientations farther away. We report direct experimental observation of a curvature-induced Dzyaloshinskii–Moriya interaction (DMI), which is quantified to be approximately one-third of the intrinsic DMI in Co/Pd stacks. The curvature induced DMI enhances stability of Néel-type domain walls. These experimental observations are further supported by micromagnetic simulations. Altogether, our findings demonstrate that introducing curvature into magnetic nanostructures provides a powerful strategy for tailoring complex magnetic behaviors, paving the way for the design of advanced 3D racetrack memory and neuromorphic computing devices.

## Graphical abstract

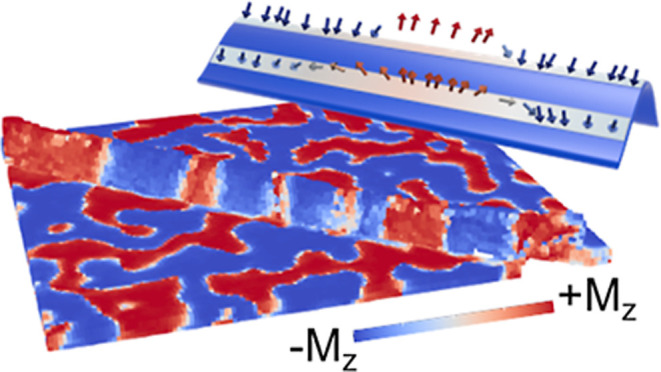

## Introduction

Recent advances in nanomagnetism are driving interest in 3D nanomagnetic systems, as extending nanomagnetism from 2D to 3D enables the exploration of more complex geometries, novel magnetic phenomena, e.g., 3D spin textures, and improved performance in spintronic applications. This could open up new opportunities to explore nanomagnetic systems with higher complexity, new functionalities and enhanced properties.^1–3^ Significant advances in modeling and theory, synthesis and fabrication, and characterization and validation are rapidly emerging to address the challenges associated with it.^4–6^

Novel synthesis methods, such as templated and cooperative growth,^7–9^ self-assembly,^10^ nanoimprinting,^11^ focused electron beam induced deposition (FEBID),^12–15^ and two-photon-lithography^16,17^ have matured and are capable of designing and fabricating tailored 3D nanostructures.^18^ These nanostructures can either be directly synthesized using such methods, or indirectly by using various thin film deposition or coating methods, such as atomic layer deposition or sputtering onto 3D scaffolds.^19^

High resolution electron microscopies have been used to study the 3D stray field distribution in nanospirals,^20^ topological magnetic fields in helical nanostructures,^21^ 3D spin textures in skyrmion tubes,^22^ vortex strings^23^ or Hopfion rings.^24^ Magnetic X-ray nanotomography has proven to be a powerful tool with unique features to obtain insight of 3D spin configurations, e.g. in topological spin textures such as skyrmions,^25^ Bloch points,^26^ vortex rings,^27^ or artificially designed nanostructures, e.g. twisted nanohelices,^15^ or 3D artificial spin ice systems.^28^

Curvature as a new design parameter for 3D magnetization textures has been proposed theoretically^29,30^ and experimentally validated in numerous systems including rolled-up nanotubes fabricated by strain engineering^31^ and magnetically capped nano- and micron sized spheres.^32–37^ The use of geometric curvature can modify magnetic interactions and local energy landscape at length scales accessible to nanofabrication and thus magnetic configurations at submicron length scales can be controlled.^5,6^ In particular, it was theoretically predicted that geometric curvature can lead to the modification of chiral magnetic interactions with an impact on magnetization textures.^38,39^ There are already first encouraging results on exploring geometric twists^40^ and bends^41^ for stabilization of homochiral domain walls, which stimulates further activities targeting observation of curvature induced effects on skyrmions^42^ and skyrmionium^43^ states.

Here, we report investigations of curvature-induced modifications of magnetic configurations in a magnetic thin film with perpendicular anisotropy deposited on an interconnected nanowire (NW) network. Interconnected magnetic NW networks are technologically interesting and have been previously studied with regard to their potential to design new types of 3D information storage and neuromorphic computing elements.^8,44,45^ In this study, the network consisting of Cu NWs with diameters of ∼50 nm and lengths up to several micron serves as 3D scaffolds for the synthesis of the curved film, and we focus on understanding the stabilization and modification of domain patterns as a function of the curvature governed by the NW network.

The random arrangement of NWs forming a network enables the investigation of both individual NWs with fixed curvature around their symmetry axes, as well as overlapping NWs, such as crossed configurations that introduce saddle points in the geometry. Therefore, such a NW network serves as a prototype for exploring various curvature classification categories, e.g., positive and negative curvatures. In this work, we first focus on understanding curvature-induced effects by studying a curved film above a single NW. Even in this simplified scenario, intriguing properties emerge. In the curved region, the magnetic easy axis aligns with the normal direction of the curved surface, resulting in deviations of the magnetization from the substrate normal. The demagnetization energy further drives the domains near the curved film to align parallel to the NW long axis. This results in preferential domain alignment in the film over the entire network. Additionally, we show that the chirality of the domain wall is influenced by the curvature-induced modification of the Dzyaloshinskii–Moriya interaction (DMI). We experimentally observe and quantify the strength of the curvature-induced DMI and demonstrate that it is approximately one-third of the intrinsic DMI in Co/Pd multilayers.

## Results/Discussion

A sketch of the experimental setup of the curved platform used for magnetic soft X-ray nanotomography is shown in Figure 1a, consisting of NW networks coated with a multilayer thin film. The networks were prepared from electrodeposited Cu NWs with ∼50 nm diameter and several microns in length.^46–48^ A thin film of Pd (7)/[Co (0.4)/Pd (0.6)]_20_/[Co (0.7)/Pd (0.6)]_20_/Ta (2.5) (all numbers in nm) was grown on the network by sputtering. Such Co/Pd multilayers exhibit strong PMA, leading to zero field stabilization of the well-known labyrinth domains with magnetization perpendicular to the substrate.^49,50^ Although the multilayer structure is nominally symmetric, the presence of a 7 nm bottom Pd layer as well as strain and defects in the multilayers can introduce a net interfacial-DMI that defines the overall chirality.^51–55^ The NWs are randomly dispersed, forming small, localized networks several microns in size, while the film uniformly covers the membrane, including regions with and without networks. This ensures the sample contains both flat regions and those with curvatures, enabling a detailed comparison of how curvature influences magnetic configurations.

**Figure 1:**
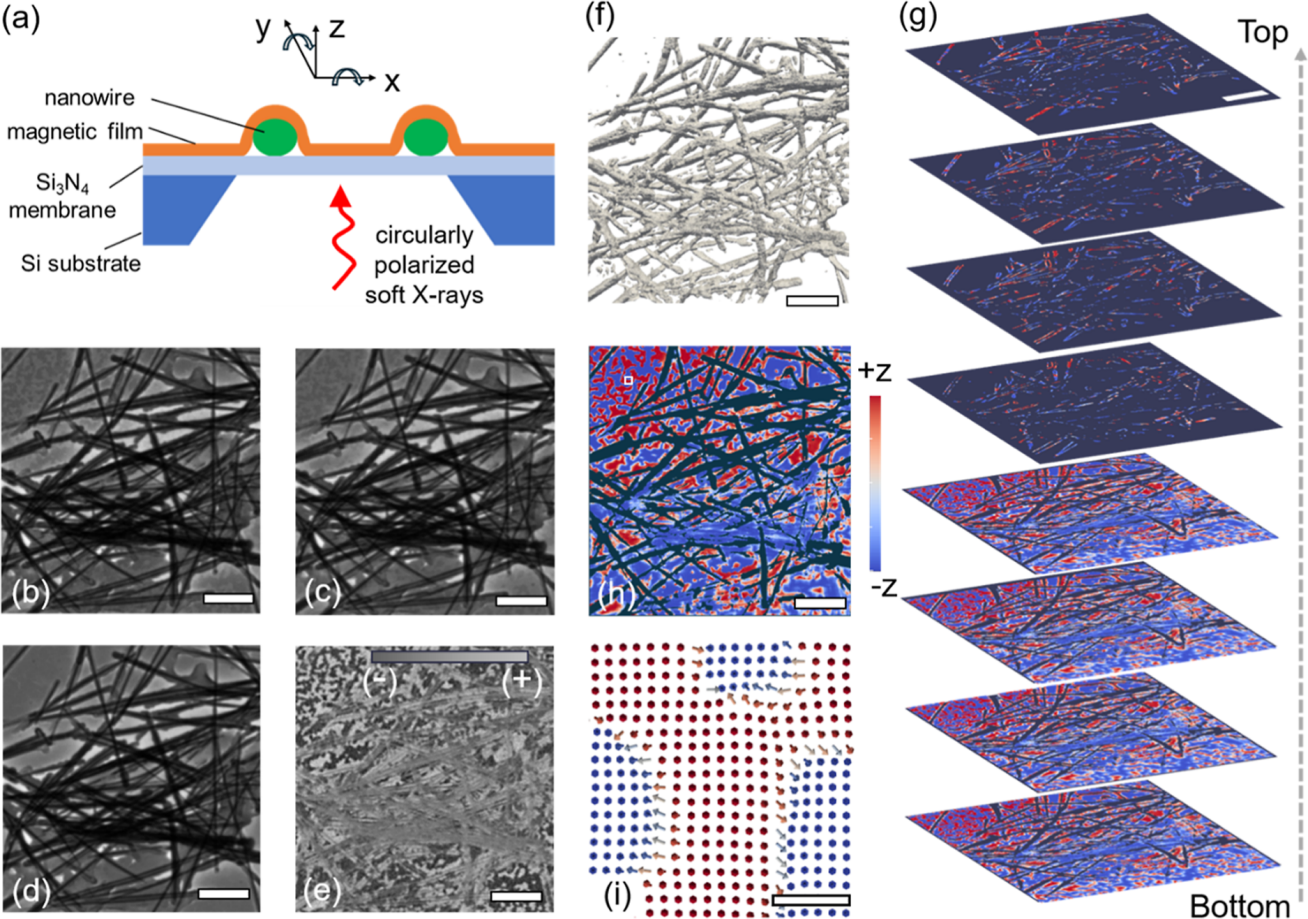
3D magnetic X-ray nanotomography of a curved thin film. (a) Schematic of experimental setup for magnetic transmission soft X-ray microscopy (MTXM) indicating the rotation around x and y axis for nanotomography, (b) right circularly polarized magnetic contrast ln (*T*_+δ_), (c) left circularly polarized magnetic contrast ln (*T*_–δ_), (d) nonmagnetic signal ln (*T*_–δ_) + ln (*T*_+δ_), (e) magnetic signal ln (*T*_–δ_) – ln (*T*_+δ_), (f) reconstructed density isosurfaces, (g) reconstructed layer-by-layer magnetic information, (h) one slice from (g) with out-of-plane magnetization represented in false color, (i) zoomed-in view of magnetization vectors in the area marked by the small white rectangle in panel (h). The scale bar is 1 μm in panels (b–h), and 54 nm in panel (i).

To visualize the 3D arrangement of magnetization, magnetic soft X-ray nanotomography was performed at the full-field soft X-ray transmission microscope at the MISTRAL beamline of the ALBA light source.^56^ Figure 1b,c show the normal incidence images with right and left circular X-ray polarization, where the transmission is denoted as *T*_+δ_ and *T*_–δ_, respectively. The nonmagnetic component was calculated by taking ln­(*T*_–δ_) + ln­(*T*_+δ_) and magnetic component (dichroism) was calculated by taking ln­(*T*_–δ_) – ln­(*T*_+δ_), as shown in Figure 1d,e, respectively.

To obtain 3D tomography of the magnetization, a tilt series of images was recorded with the sample tilted around the *x*-axis in the plane of the membrane (along one edge of the membrane) from −54° to +54° in 2° increments [Figure 1a]. A second tilt series was then acquired after rotating the sample 90° about the *z*-axis (perpendicular to membrane), which was equivalent to tilting the sample around the *y*-axis shown in Figure 1a. 3D reconstructions were generated using an open-source iterative tomography solver^57,58^ with an effective voxel size of ∼9 nm and a half-pitch spatial resolution of ∼30 nm (see Supporting Information for details). The structural density is shown in Figure 1f, confirming that the NW network structure is accurately captured, with the 3D stacking of wires clearly visible in the reconstruction. Figure 1g presents the magnetic information across different layers of the curved film deposited onto the NW network, demonstrating the ability to reconstruct magnetization along the *z*-direction. One such layer is shown in Figure 1h using false color to represent the *z*-component of the magnetization. This planar cut is in good qualitative agreement with the normal-incidence projection image [Figure 1e]. Figure 1i shows a zoomed-in view of the magnetic reconstruction of the area marked by the white rectangle in Figure 1h, where magnetization vectors illustrate the capability to resolve full 3D magnetization information. Thus, tomography provides access to spatially resolved 3D magnetic configurations within the volume. Although, the reconstruction is performed over the full 10 μm field of view, we will start in the following with a focus on the curved film above a single NW to obtain a deeper understanding of geometry induced effects in this simpler case.

### Curvature-Induced Variation in Anisotropy Direction

In Figure 2a, we show the experimental full vector reconstruction of a curved section of the film delineated on a NW. Both the planar and curved regions of the film exhibit out-of-plane magnetic domains. The *x*-component of the magnetization configuration is shown in Figure 2b, where a pronounced difference is observed between the domain pattern in the planar and curved regions. In the planar region, the *x*-component merely demarcates the boundaries of the domains. However, in the curved region, the domains themselves exhibit a significant *x*-component with the contrast alternating between positive and negative values shown by red and blue colors, respectively. Furthermore, by examining the top view of both the *z*-component [Figure 2c] and the *x*-component [Figure 2d] of the magnetization in the curved region over the NW, we find that the contrast alternates within each domain along the NW. To aid visualization, the domains on the curved section are outlined with dashed lines and rectangles, with the ridge line of the NW marked with a solid line. For example, in the top-right domain 1 oriented along the +*z* direction, the *x*-component contrast is positive along the right side of the domain (red) and negative on the left side (blue). The adjacent domain 2, oriented along −*z* direction, shows the opposite behavior, with the *x*-component contrast negative on the right (blue) and positive on the left (red). This alternating contrast can be observed for most of the domains on the curved part. It demonstrates that in the curved region, the magnetization is not strictly out-of-plane within each domain as observed in the planar region, rather it fans out toward the local normal of the curved surface. This points to a possible modification of the anisotropy direction or the magnetostatic energy by the curvilinear geometry.

**Figure 2:**
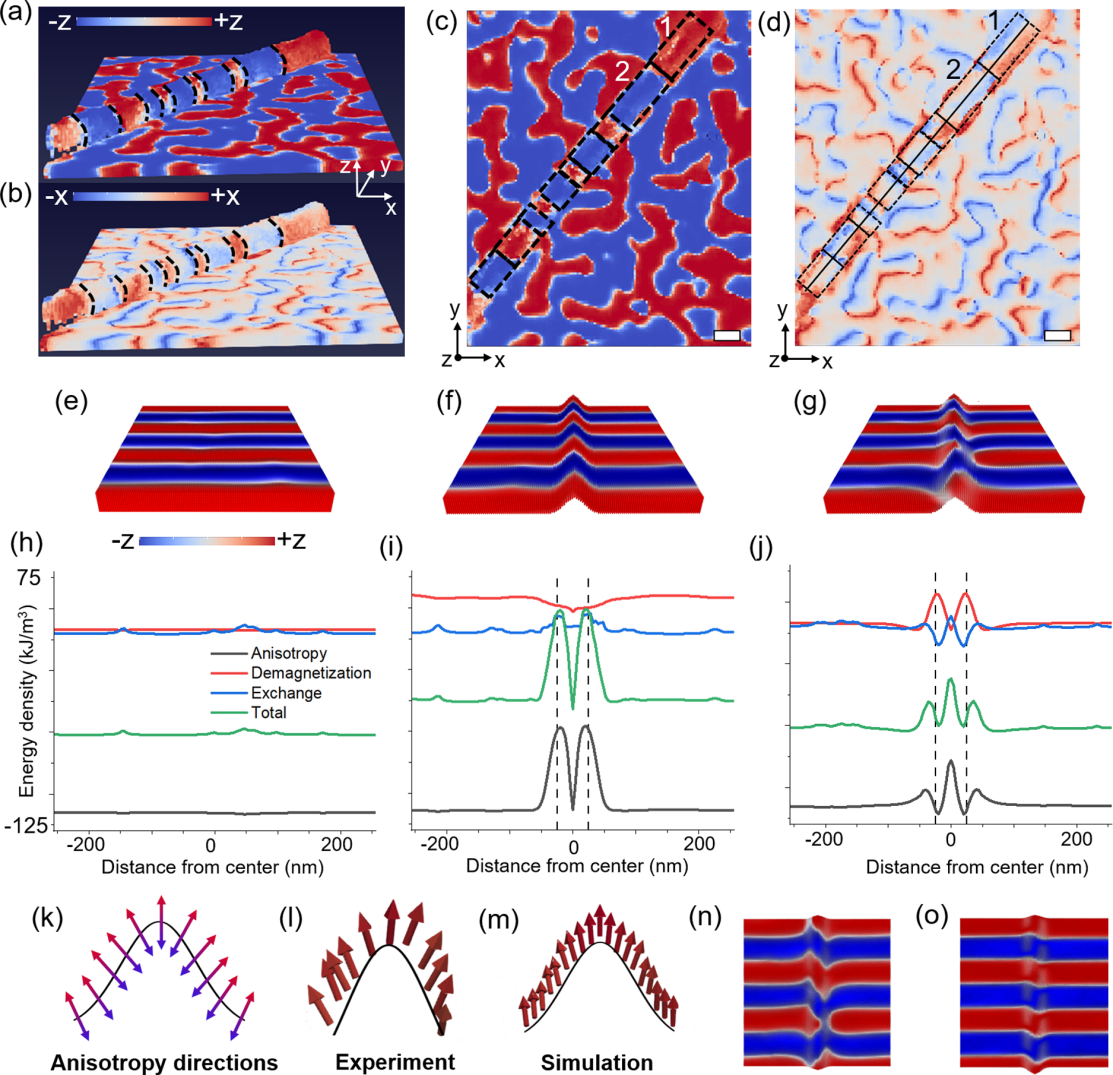
Curvature induced anisotropy. Side and top view of (a,c) the *z*-component and (b,d) the *x*-component of the magnetization in the curved film, respectively. The dashed lines and rectangles highlight the domains in the curved region. The solid line in panel (d) marks ridge line of the NW. The lateral dimensions are 1080 nm × 1530 nm. Scale bars in panels (c,d) are 100 nm. (e–g) Micromagnetic configuration of a 512 nm × 512 nm area and (h–j) corresponding energy densities across the simulated volume for the (e,h) planar film with stripe domains, and curved film (f,i) before and (g,j) after relaxation of stripe domains, respectively. The dashed lines in panels (i,j) show the width of the Gaussian used in the simulation. (k) Schematic of anisotropy vector orientation conforming to the curved surface, (l,m) magnetization vectors along a line cut from experiment and simulation, respectively. (n,o) Top view of the micromagnetic configuration with and without considering the demagnetization energy in the simulation, respectively.

To understand this, a curved film over a single nanowire is simulated using Mumax3 with parameters typical of Co/Pd multilayers:^59^ saturation magnetization *M*_s_ = 500 kA/m, anisotropy constant *K*_u_ = 0.15 MJ/m^3^, exchange stiffness *A*_ex_ = 10 pJ/m.^53,60^ The total mesh size is 1020 nm × 1020 nm × 168 nm with a cell size of 4 nm × 4 nm × 4 nm. The thickness of the film is considered to be 52 nm. Further details of the simulation setup are provided in the Supporting Information. First, stable stripe domains with moments aligned in neighboring domains with alternating out-of-plane magnetization are formed by energy minimization in a planar film [Figure 2e]. These domains are then mapped onto a curved geometry defined by a Gaussian profile, and relaxed to minimum energy state. When the magnetic easy axis is assumed to be along the ±*z* directions, the magnetization orientation does not rotate toward the local normal of the curved surface after relaxation [Figure S2]. However, when the magnetic easy axis is set to be locally normal to the curved surface [Figure 2k], the stripe domains in the planar regions remain largely unchanged and those on the curved surface exhibit a reduced *z*-component after relaxation, similar to the experimental observations [Figure 2l,m]. The magnetization states before and after relaxation are shown in Figure 2f,g. Thus, curvature induced modification of the magnetic easy axis is a necessary condition to reproduce the experimental observations. This is in line with earlier observations in Co filaments,^61^ in Co/Pd and Co/Pt caps on spherical particles^33,35^ and in rolled-up architectures.^31,62^ Interestingly, the magnetization does not fully align with the new easy axis, i.e., the local normal of the curved surface [Figure 2k–m], possibly due to large exchange penalty.

Different micromagnetic energies for the three cases shown in Figure 2e–g are compared in Figure 2h–j. Energies are evaluated as a function of the distance from the center of the simulated region, in the direction perpendicular to the ridge line of the curved surface. In the planar film case, the energies are constant across the film. Mapping the stripe domains onto the curved geometry leads to an increase in the total energy [Figure 2i]. This can be attributed to two main factors: (1) curvature-induced variation in the magnetic easy axis—the anisotropy energy peaks at the edges of the curved region, where the magnetization deviates most from the nominal anisotropy direction, and (2) enhanced demagnetization effects. After relaxation, the anisotropy energy is decreased as the magnetization rotates to follow the easy axis [Figure 2j, black curve]. Furthermore, the stripe domains tend to bend away from the top of the curved surface and in some instances, they split into two branches [Figure 2n]. This allows minimization of the magnetostatic energy [Figure 2j, red curve], which favors magnetization alignment along the long axis of the underlying NW. Simulations without the magnetostatic term does not show such alignment (Figure 2o). Thus, due to the magnetostatic energy of the curved system, domains prefer to align parallel to the sides of the curved surface, rather than spanning directly over it. This tendency may trigger a pronounced effect on the magnetic domains in the planar region adjacent to the curved region, which we investigate next.

### Influence of Curvature on Domain Wall Orientation

To correlate the magnetic domains with the network morphology, we explore the film over the full network beyond just a single NW and examine their alignment across the entire field of view of 10 μm. To describe and quantify the effect of curvature on the domain wall formation, in our tomographic analysis we employ the formalism of differential geometry.^63^ There, a two-dimensional surface at each point is characterized by two principal curvatures *K*_1_ and *K*_2_. The mean curvature is the arithmetic mean of those principal curvatures $H=\frac{K_{1}+K_{2}}{2}$ and the Gaussian curvature is the square of the geometric mean of *K*_1_ and *K*_2_, *K* = *K*_1_ *K*_2_. For a nanowire geometry, one of the principal curvatures (along the wire) is zero. Details of curvature analysis is provided in the Supporting Information. With the combined magnetic and curvature maps, it is possible to investigate correlations between the images.

For this purpose, magnetic domains are binarized by selecting a threshold at |*M*_*z*_| > 0.7, where *M*_*z*_ is the *z*-component of the normalized magnetization, and structural features are binarized by thresholding the map of the principal curvature *K*_1_ at >0.4 [Figure 3a,b]. The map of principal curvatures is calculated based on the analysis of the transmitted intensity as discussed in Supporting Information Section SI5 [Figure S4]. From the binary maps, ellipse fitting extracts the orientation, positions and size of the magnetic and curvature regions. The orientation of the domain walls and the NWs can be quantified as the angle between the major axes of the fitting ellipses. We find that at distances close to the NWs, the domains are oriented more along the axis of the wire (e.g., an average angle ∼ 33° for 100 nm), and at distances far from the NW (1000 nm), the domains are oriented at random angles with an average angle of ∼45° [Figure 3c]. The angle between NW and domain is taken for each domain as the angle with the nearest NW. The statistics are collected for all rotation angles. For each radial distance the domains are binned into 100 nm wide segments to define a population of domains. For each population of domains, a histogram is generated [Figure 3d]. The histograms are skewed toward 0°, which is the parallel orientation of domain and nanowire. For larger distances the average angle converges to 45° which is expected for a population of randomly oriented line segments.

**Figure 3:**
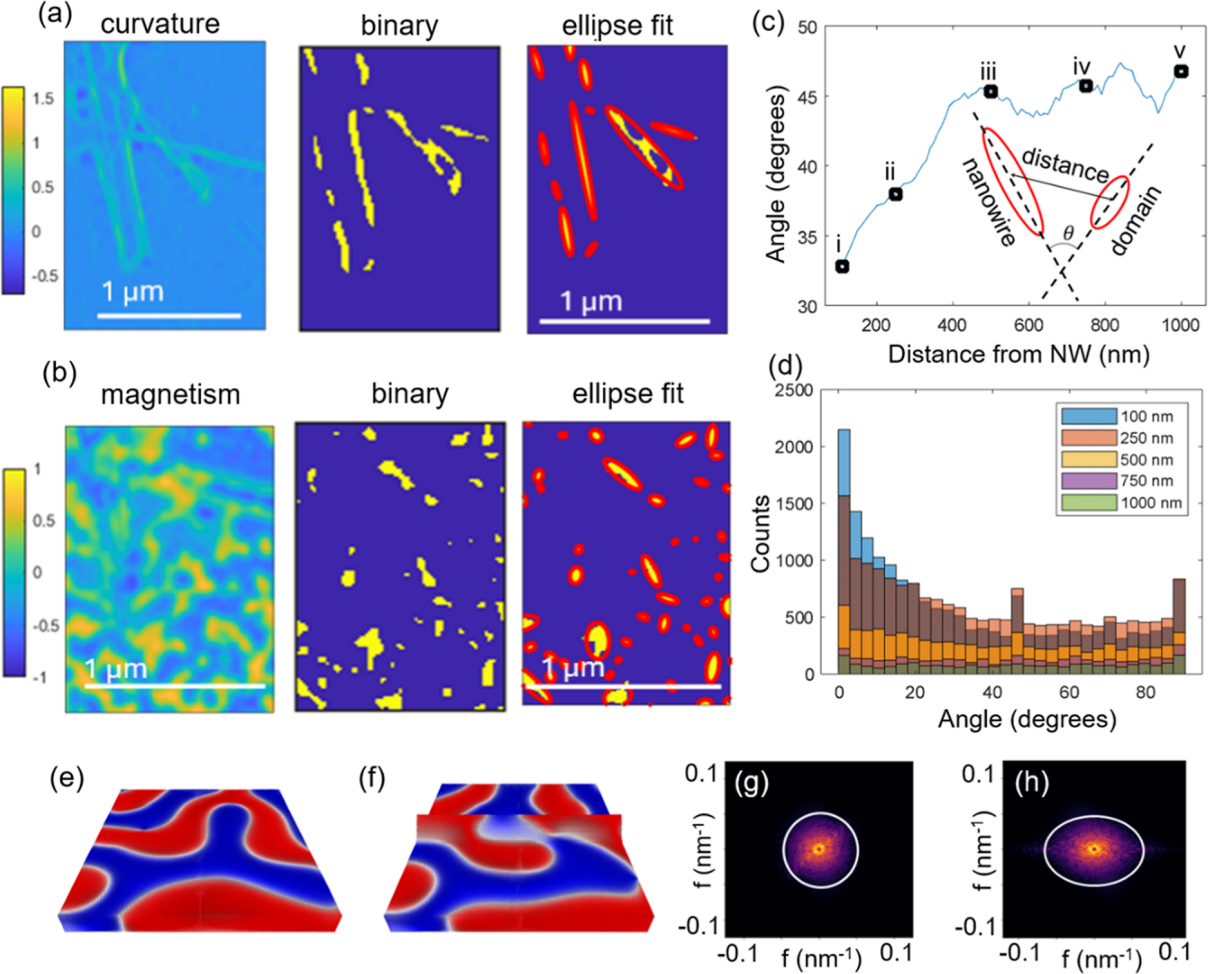
Curvature induced domain alignment. (a) Representative field of view of the map of principal curvature *K*_1_, its binarized image (thresholded at *K*_1_ > 0.4) and ellipse fitting of the binarized image. (b) Same region in magnetic domains, binary segmentation selecting greatest magnetic contrast and ellipse fitting of regions of greatest magnetic contrast, where the magnetic domains are selected as the region with |*M*_*z*_| > 0.7. (c) Ellipse fitting of domains at different distances from NWs, from 100 to 1000 nm (i–v). (d) Plot of mean from histograms showing average angle between NW and magnetic domain as a function of separation corresponding to the distances labeled (i–v) in (c), converging near 45° beyond 400 nm separation. (e,f) Micromagnetic configuration in a 512 nm × 512 nm area and (g,h) Fourier transform of the domain pattern in the planar and the curved geometry, respectively.

Micromagnetic simulations are performed to verify these effects using labyrinth domains, as observed experimentally. Figure 3e shows that a typical labyrinth domain pattern stabilizes in a planar film upon relaxation from a random magnetization state. Using the same random seed and introducing curvature, a clear difference is found both on the curved region and in the adjacent planar areas [Figure 3f]. For example, the orientation of the central domain remains random in the planar region, but on the curved region, it expands along the ridge line. Additionally, this reorientation influences neighboring domains; the domain at the edge becomes more aligned with the ridge line of the curved surface [Figure 3f]. These qualitative observations are further examined using Fast Fourier Transform (FFT) analysis. Simulating domain relaxation in planar and curved films across 20 different random magnetization seeds reveals a consistent trend. For the planar film, a ring structure in the FFT [Figure 3g] indicates uniform domain periodicity with random orientations. In contrast, when the domains from the curved structure are mapped onto a 2D plane preserving spatial relationships for the FFT analysis, a distinct alignment can be seen along the ridge line, evident from the ovular shape in Figure 3h. Hence, the geometry induces domain realignment by guiding the magnetization to follow the principal direction with zero curvature, leading to preferred orientations along the curved contours. Micromagnetic simulation shows that the magnetization reversal under external field is also strongly influenced by such curvature induced domain alignment (Figure S3).

### Impact of Curvature on Chirality of Domain Walls

Lastly, the effect of curvature on the chirality of domain walls (DWs) is examined. The DW chirality is characterized by measuring the angle between the DW magnetization (**m**) and DW normal vector (**n**) [Figure 4a inset].^52^ The area shown in Figure 2a is analyzed, consisting of 120 pixels × 170 pixels with dimensions 1080 nm × 1530 nm. In the planar region, a predominance of right-handed Néel walls is observed as shown in the histogram [Figure 4a], a manifestation of the DMI in the system. However, a significant number of DWs deviate from the purely right-handed Néel type. In contrast, in the curved part of the film, right-handed Néel DWs are more prevalent, as indicated by the tighter distribution in the histogram [Figure 4b]. Magnetization profile of two DWs, one from the top and one from the side of the curved region are shown in Figure 4c. For the DW located at the top, the magnetic easy axis is completely out-of-plane while demagnetization energy favors in-plane alignment of magnetic moments parallel to the long axis of the NW (now defined as the *x*-axis in Figure 4). As a result, a right-handed Néel DW is stabilized and characterized by a strong positive *x*-component and minimal *y*-component of magnetization (in the base plane and orthogonal to *x* direction) while the normalized *z*-component switches between ±1 [Figure 4d]. At the sides of the curved region, a more complex behavior seems to emerge. As discussed earlier, magnetization in these regions tends to fan out to align with the local surface normal. This gives rise to domain walls with distinct characteristics, where the DW exhibits a sizable *y*-component and a reduced *z*-component contrast, transitioning between approximately ±0.75 rather than fully switching between ±1 [Figure 4e]. This resembles a twisting of the domain wall structure. On the other side of the curved surface, the twisting seems to be in the opposite direction with a reversed *y*-component [Figure S5]. However, if expressed in a coordinate basis rotated in the *yz* plane to align with the direction of maximum |*M*_*z*_|, represented by *x*_R_ – *y*_R_ – *z*_R_ in Figure 4c, these walls also correspond to Néel DWs (Figure 4f).

**Figure 4:**
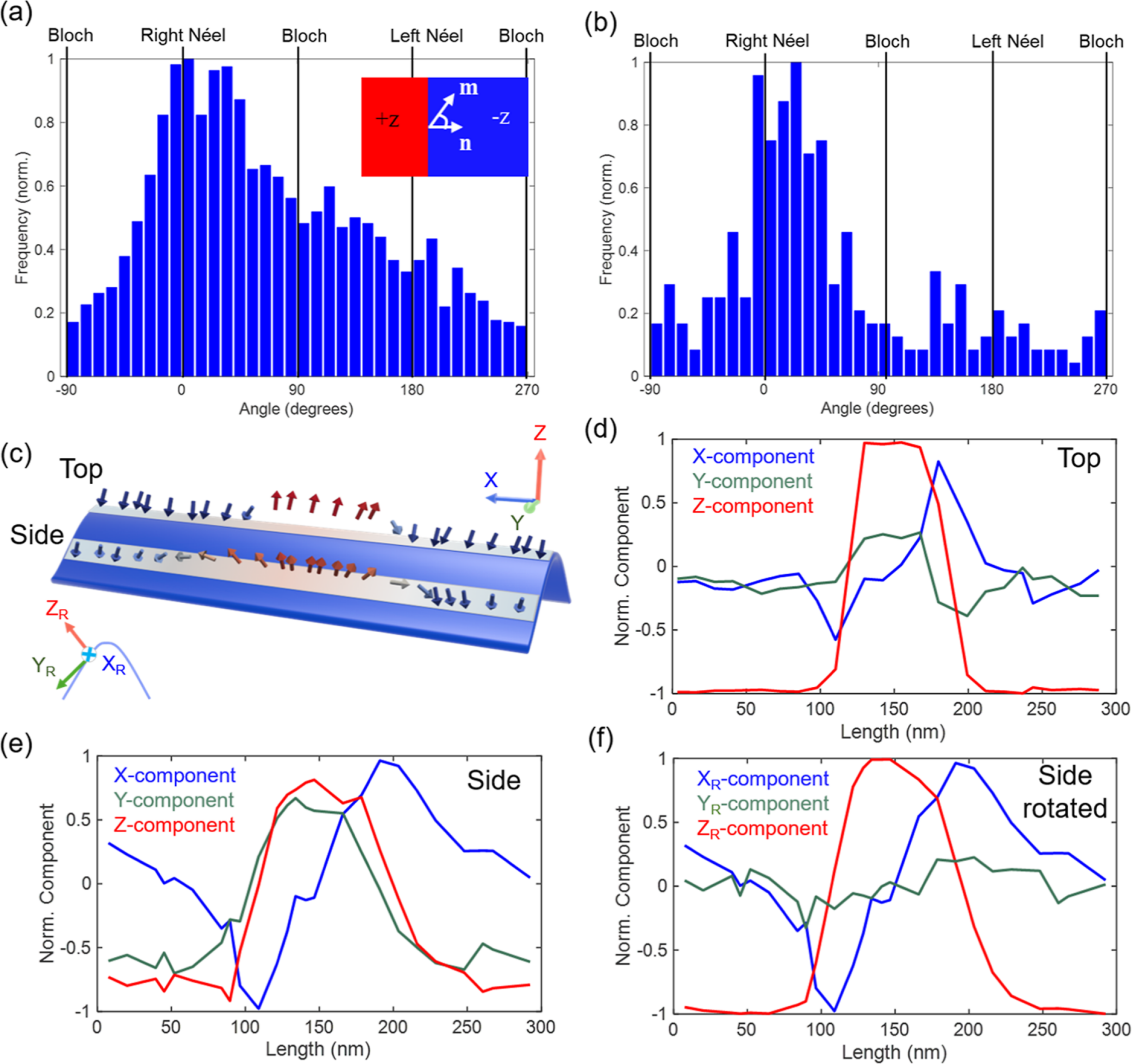
Curvature modified chirality of domain walls. Histograms of angle between DW magnetization and DW normal vector, measured pixel-by-pixel along the DW centerline in (a) the planar and (b) the curved region of the film, respectively. (c) Schematic illustration of the experimentally observed magnetic configurations (d) on top and (e,f) side of the curved region, in (d,e) the *x* – *y* – *z* and (f) *x*_R_ – *y*_R_ – *z*_R_ coordinates.

Therefore, curvature strongly influences domain wall chirality, promoting Néel-type walls. This finding is indicative of the additional contribution stemming from the curvature induced DMI, which adds to the intrinsic interfacial DMI in Co/Pd stacks. From the histograms, we estimate the fraction of right-handed Néel DWs as $[(\sum_{-45^{{}^{\circ}}}^{{+45}^{{}^{\circ}}}counts$ / $\sum_{-90^{{}^{\circ}}}^{{+270}^{{}^{\circ}}}counts)$ × 100%], where 0° corresponds to purely right-handed Néel DWs. This fraction is found to be 40.8% in the planar region and 53.9% in the curved region. This 13.1% enhancement suggests that curvature contributes an effective DMI approximately one-third the strength of the intrinsic DMI in Co/Pd stacks. It can be attributed to the magnetostatic energy which favors alignment parallel to the long NW axis similar to cylindrical magnetic NWs. This gives rise to the strong magnetization component along that direction and consequently more right-handed Néel DWs are stabilized. Theoretically, the magnitude of such curvature induced DMI is calculated as *D*_c_ = 2*A* × curvature,^30^ where curvature for the case of a cylindrical surface is 1/*R*, with *R* the radius of cylinder and *A* the exchange constant. For the geometry of our NWs (*R* = 25 nm) and assuming *A* = 10^–11^ J/m, we obtain *D*_c_ = 0.8 mJ/m^2^. Additionally, strain gradients induced by curvature and increased roughness from the NWs could also enhance DMI.^64–66^

DMI in Co/Pd multilayers have been measured previously. Even in symmetric multilayers, significant DMI can arise due to unequal strain from the bottom Pd/Co and top Co/Pd interfaces.^54^ For a single Pd/Co interface, DMI is typically positive.^51,52^ The sign of DMI in multilayers depends on Pd layer thickness,^54^ and in trilayers it was reported to vary between ±0.3 mJ/m^2^. In our case, the sign of DMI was negative as indicated by the right-handed Néel DWs. The DMI strength also strongly depends on the number of Co/Pd bilayers and has been shown to increase 3-fold (from ∼1 mJ/m^2^ to ∼3 mJ/m^2^) when repetition number increases from 1 to 20. Therefore, a significant intrinsic DMI of up to 3 mJ/m^2^ can be expected in our system, and the contribution from the curvature-induced DMI estimated at 0.8 mJ/m^2^, would be roughly one-third of the intrinsic DMI as assessed based on the analysis of the histograms in Figure 4. To our knowledge, this is the first experimental quantification of the curvature induced DMI in perpendicularly magnetized curved thin films. These results will motivate future experiments to quantify DMI in curved magnetic films using alternative methods such as asymmetric domain wall propagation and Brillouin light scattering.^67^ Furthermore, in contrast to prior observations in in-plane systems,^41^ our results pave the way to experimental search of curvature stabilized topological spin textures.^42^ These findings will also open new avenues for 3D curved racetrack memory.^3,40^

## Conclusions

In summary, we have investigated the effect of curvature on magnetic configurations in a Co/Pd multilayer film with PMA coated on a NW network scaffold. The sample was measured with soft X-rays with circular polarization for magnetic contrast. The data were reconstructed in 3D using vector-resolved nanotomography and the domain structure was found to be continuous across the planar and curved parts of the sample. The curved geometry modifies the orientation of the easy axis and the magnetization follows the local normal of the curved surface. The correlation between domain structure and curvature is quantified by measuring the orientation of the domains in the sample as a function of separation to the nearest NW which shows parallel alignment at small distances and random alignment at larger distances. Furthermore, our work experimentally confirms the theoretical concept of curvature induced DMI in PMA thin films and paves the way toward stabilization of curvature induced skyrmions and skyrmionium states. Micromagnetic simulations support our experimental findings, confirming that the energy landscape is modified on the curved surface. This work demonstrates that curvature modifies the local magnetic easy axis, domain structure and chirality of domain walls, offering an effective method to tailor spin textures and energy landscapes at the nanoscale.

## Methods/Experimental

### Sample Fabrication

Cu NWs with 50 nm diameter and several microns in length were first prepared by electrodeposition into nanoporous polycarbonate membranes. The NWs were harvested by dissolving the membrane in dichloromethane, then liquid-exchanged with deionized water before being drop-cast onto a 50 nm thin silicon nitride (Si_3_N_4_) membrane. The Si_3_N_4_ membrane allows for sufficient transparency in the soft X-ray regime required to perform magnetic transmission soft X-ray microscopy (MTXM). The Co/Pd film was grown on the network by DC magnetron sputtering under a base pressure below 3 × 10^–8^ Torr and an Ar working pressure of 5 mTorr.

### Magnetic X-ray Nanotomography

For magnetic soft X-ray nanotomography, images were recorded at the Co *L*_3_ edge with both left and right circular polarization, divided by the incident intensity to obtain the transmission, and subtracted from each other to reduce the nonmagnetic background in the magnetic images. To obtain 3D tomography of the magnetization, a tilt series of images rotated around the x and y axis was recorded and reconstructed using an iterative algorithm. The input to the reconstruction algorithm is an aligned and normalized tilt series containing both magnetic and nonmagnetic information. Using the aligned image stack, the algorithm reconstructs the structural and magnetic information in a 3D volume.

## Supplementary Material



## Notes

Details of reconstruction, micromagnetic simulation setup, micromagnetic simulation without anisotropy direction modification, micromagnetic simulation with applied field and temperature, curvature analysis and curvature induced chirality (PDF)
