# Supplementary material for: Curvature Induced Modifications of Chirality and Magnetic Configuration in Perpendicular Films
Source: ACS Nano. 2025 Aug 11;19(35):31609–18. doi: 10.1021/acsnano.5c08926 (PMC12424293; doi:10.1021/acsnano.5c08926)
Supplement: Supplementary file 1 [file nn5c08926_si_001.pdf]

Supporting Information

Curvature induced modifications of chirality and magnetic configuration in perpendicular films

David Raftrey[1,2,*], Dhritiman Bhattacharya[3,*], Colin Langton[3,*], Bradley J. Fugetta[3], Subhashree Satapathy[1], Olha Bezsmertna[4], Andrea Sorrentino[5], Denys Makarov[4], Gen Yin[3], Peter Fischer[1,2§], Kai Liu[3,#]

[1]Materials Sciences Division, Lawrence Berkeley National Laboratory, Berkeley, CA 94720, United States

[2]Department of Physics, University of California Santa Cruz, Santa Cruz, CA 95064, United States

[3]Department of Physics, Georgetown University, Washington, DC 20057, United States

[4]Helmholtz-Zentrum Dresden-Rossendorf e.V., Institute of Ion Beam Physics and Materials Research, 01328 Dresden, Germany

[5]Alba Light Source, MISTRAL beamline, 08290 Cerdanyola del Vallès, Spain

** Equal Contribution*

Email: § PJFischer@lbl.gov, # Kai.Liu@georgetown.edu

SI 1. Details of reconstruction

The relation between the transmitted intensity, the incident intensity and the magnetization vector are governed by the Beer-Lambert law:

$$I = I_0 exp(-1)\{\int \mu(t)[1 + \delta(\boldsymbol{\kappa} \cdot \boldsymbol{m})]\mathrm{d}t\}$$

Here I and I_0 are the transmitted and incident intensities of the X-ray beam, respectively. μ is the linear absorption coefficient, δ is the dichroic coefficient which characterizes the sensitivity of the material's X-ray magnetic circular dichroism (XMCD) effect and depends on the electronic levels of the absorbing atoms. $\boldsymbol{\kappa} \cdot \boldsymbol{m}$ is the dot product of the X-ray wavevector and magnetization with $\boldsymbol{m}$ the reduced magnetization vector ($\mathbf{m} = \mathbf{M}/M_S$, with $\mathbf{M}$ the magnetization vector and M_S the saturation magnetization) and dt is the differential path element along the X-ray trajectory spanned by the line integral. The total transmission is therefore the sum of two terms:

$$T = \frac{I}{I_0} = exp(-1)\{\int \mu(t) \cdot dt + \int \mu(t) \cdot \delta(\boldsymbol{\kappa} \cdot \boldsymbol{m})\}$$

The first term is purely non-magnetic (charge absorption), and the second term encodes the magnetic contribution due to XMCD. By switching between right-handed (+δ) and left-handed (−δ) circular polarization in the experiment, we measure two transmission values:

$$T_{+\delta} = exp(-1)\left\{\int \mu(t) \cdot dt + \int \mu(t) \cdot \delta(\boldsymbol{\kappa} \cdot \boldsymbol{m})\right\}$$

$$T_{-\delta} = exp(-1)\left\{\int \mu(t) \cdot dt - \int \mu(t) \cdot \delta(\boldsymbol{\kappa} \cdot \boldsymbol{m})\right\}$$

For practical purposes, in order to remove the exponential, we take the natural logarithm of both of them to obtain the corresponding "absorbance":

$$-ln[T_{+\delta}] = \int \mu(t) \cdot dt + \int \mu(t) \cdot \delta(\boldsymbol{\kappa} \cdot \boldsymbol{m})$$

$$-ln[T_{-\delta}] = \int \mu(t) \cdot dt - \int \mu(t) \cdot \delta(\boldsymbol{\kappa} \cdot \boldsymbol{m})$$

And finally, to separate the magnetic and the non-magnetic contribution we add and subtract ln $[T_{-\delta}]$ and $ln[T_{+\delta}]$:

$$ln[T_{-\delta}] + ln[T_{+\delta}] = -2\int \mu(t) \cdot dt$$

$$ln[T_{-\delta}] - ln[T_{+\delta}] = 2\int \mu(t) \cdot \delta(\boldsymbol{\kappa} \cdot \boldsymbol{m})$$

The second equation is what we called "magnetic signal" and it is linearly proportional to the projection of the magnetization along the X-ray direction, weighted by the local absorption coefficient and dichroic sensitivity.

Circularly polarized X-rays with opposite degree of polarization are emitted above and below the orbital plane from the bending magnet. In order to switch the degree of polarization illuminating the specimen the electron bunches inside the storage ring are deflected at the MISTRAL source point [1] to locally change the direction of the orbit plane. Flat field images are also taken off-sample to obtain an image of the beam profile for further image corrections.

Raw data collected is the CCD intensity of both the transmitted signal through the sample (I) and flat field signal taken away from the sample I_o. The X-ray transmission T is then calculated as $T=I/I_o$. The transmitted signal for each polarization contains small asymmetries originating in imperfect optical properties of the beamline. To account for these asymmetries to first order, the transmitted intensities for each pixel ij are normalized over all angles Θ by $T_{norm} = \frac{T_{ij}^{\theta}}{\sum_{ij} T_{ij}^{\theta}}$. From the transmitted signal the magnetic component is calculated by taking $ln[T_{-\delta}] - ln[T_{+\delta}]$ and the non-magnetic component is calculated by taking $ln[T_{-\delta}] + ln[T_{+\delta}]$ as discussed earlier.

To account for sample stage drift during the acquisition of the data, an iterative alignment procedure is implemented. The two main classes of image alignment algorithms are feature recognition and cross correlation. Cross correlation presents challenges for aligning images with different contrast due to the opposite XMCD contrast. In this case we begin with an alignment by feature recognition picking out high derivative areas i.e. corners, or defects implemented in MATLAB©. This feature recognition method aligns the left and right polarized image stacks. From the aligned single polarization images the difference is taken to compute the magnetic signal, and the sum is taken to compute the non-magnetic structural contrast. Regions of bright pixels where there are holes in the membrane are masked out of the image processing. Finally, a cross-correlation alignment is applied to the stack of images with XMCD contrast, now that the difficulty of the alternating contrast from left vs right polarization has been resolved by taking the differences. The vector-resolved tomography solver takes as input images with combined signals from magnetism and structure. The magnetic contribution to the image is scaled to be equal to the structural contribution to the image. Finally, we compute a second image stack with a spatial inversion and a reversal in the order of subtraction to create a compatible stack of twice the angular range mirrored around the rotation axis with $ln[T_{-\delta}] - ln[T_{+\delta}]$ for $\Theta =$ -54° to 54° and $ln[T_{+\delta}] - ln[T_{-\delta}]$ for $\Theta =$ 126° to 234° for the mirrored stack (180°± 54°). This ensures that the magnetic signal is not interpreted as structural contrast by the algorithm. The computed spatial

inversion about the axis is equivalent to having an extended angular range that extends beyond the tilt range of the stage.

Once the two polarization images are converted into transmittance images, they are aligned to one another manually for each angle using Tomviz software by observing when the structural information of the nanowires (NWs) is minimized. Furthermore, the drift in the sample throughout the series is corrected by manually aligning a particular structural feature across all images for both tilt series. This effectively creates a fixed point from which all rotations and positions are registered.

After the preliminary steps of processing and aligning all the images from the two tilt series recorded with the sample tilted from –54° to +54° in 2° increments are complete, the reconstruction of the non-magnetic structure is performed. This is done using a mesh of 2048×2048×256 voxels, where each voxel has a volume of (~9 nm)^3, matching the pixel size of the images. The method of iterative backpropagation is used for the structural reconstruction, implemented in C++ and performed for 55 iterations. This process provides a 3D mesh of the X-ray absorption density of the sample, allowing for the identification of the Cu NWs, which are far more absorptive than the magnetic film or substrate.

To create the 3D magnetic mesh, a mask of the spatial distribution of magnetic material is generated to enable the backpropagation algorithm. This mask is created by selecting a threshold density value from the structural mesh and treating it as the surface of the NWs onto which magnetic material is deposited. Approximately 40 nm of magnetic material is deposited on the sample; therefore, the four voxels above each surface voxel are included in the mask. To determine the vertical location of the magnetic film beneath the NWs, small fluctuations in the structural mesh are observed in regions of interest off the NWs, and the slice at which the fluctuations are most in focus is treated as the location of the base adhesion layer. The four voxels above this slice are also included in the magnetic mask.

Once the mask is generated, it is used along with the magnetic contrast images to reconstruct the 3D magnetic mesh. This reconstruction is performed using iterative backpropagation, similar to the structural reconstruction, but only for five iterations.

SI 2. Micromagnetic Simulation Setup

In the simulations, the film had a grid size of 255×255×42 with a cell size of 4nm × 4nm × 4nm, resulting in a total mesh size of 1020nm×1020nm×168nm. The thickness of the film was considered to be 52 nm. The saturation magnetization (M_s) was set to 500 kA/m, with an anisotropy of K_u=0.15 MJ/m³, an exchange constant of A_{ex}=10 pJ/m, and a damping parameter $\alpha = 0.3$ to ensure fast relaxation times. For the sake of simplicity, the DMI contribution was not included in the simulation parameters.

The structure was defined in Mumax3 pointwise by looping over cell indices using the equation for a Gaussian:

$$z = d * e^{\frac{-(y-y_0)^2}{w^2}}$$

where z is the vertical cell index, d is the maximum height of the curve, y is the horizontal cell index, y_0 is the center of the gaussian (127 for a 255 cell wide square) and $2w$ is the width of the Gaussian. 13 Gaussians were stacked on top of one another to simulate a 52 nm thick film (Fig. S1).

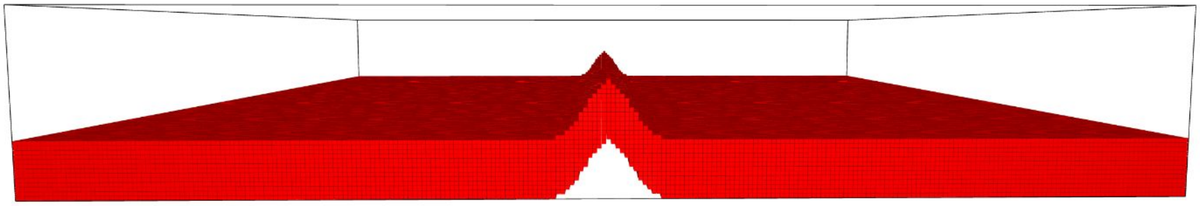

Figure S1. The simulated geometry in Mumax3.

Anisotropy was assumed to be always normal to the film and thus could be defined by the normal of the Gaussian. This was found by taking the gradient and then defining the normal vector.

$$z' = \frac{-2d(y-y_0)}{w^2} e^{\frac{-(y-y_0)^2}{w^2}}$$

$$mag = \sqrt{z'^2 + 1}$$

$$\overrightarrow{K_u} = |K_u| * (0, \frac{-z'}{mag}, \frac{1}{mag})$$

SI 3. Micromagnetic Simulation without Anisotropy Direction Modification

Micromagnetic simulations are performed with and without modification of the anisotropy direction to decouple the effect of magnetostatic and anisotropy energy. The first case is shown in the main text. Fig. S2 shows the latter case. These magnetic states are different than in the experiments, establishing that curvature-induced modification of the anisotropy direction is a necessary assumption to reproduce the experimental observations.

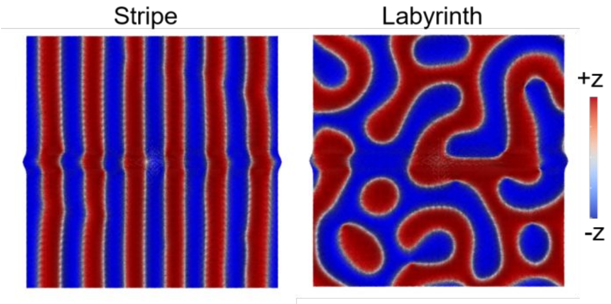

Figure S2. Energy minimized states starting from stripe and labyrinth domains when anisotropy direction was strictly out-of-plane.

SI 4: Micromagnetic Simulation with Applied Field and Temperature

Figure S3 shows the reversal process starting from a labyrinth domain state for T = 0 K and T=300 K. As the field increases, domains near the curved region begin to align along the principal direction with zero curvature (i.e., the long axis of the NW). This again demonstrates that curved regions guide the domain alignment. With further increase in the field to 3000 Oe, only the domains on the curved region persist, while those on the planar part are annihilated. This occurs because the anisotropy axes in the planar region are not aligned with the applied field. Thus,

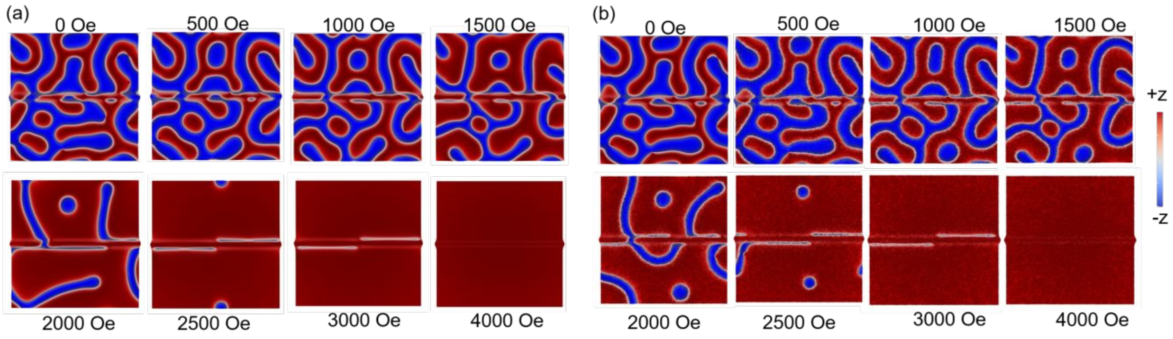

Figure S3. Magnetization reversal starting from a labyrinth state at (a) T=0 K and (b) 300 K.

curvature-induced modification of magnetic textures, as seen in experiments, strongly influences the magnetization reversal behavior under applied magnetic fields.

SI 5. Curvature analysis

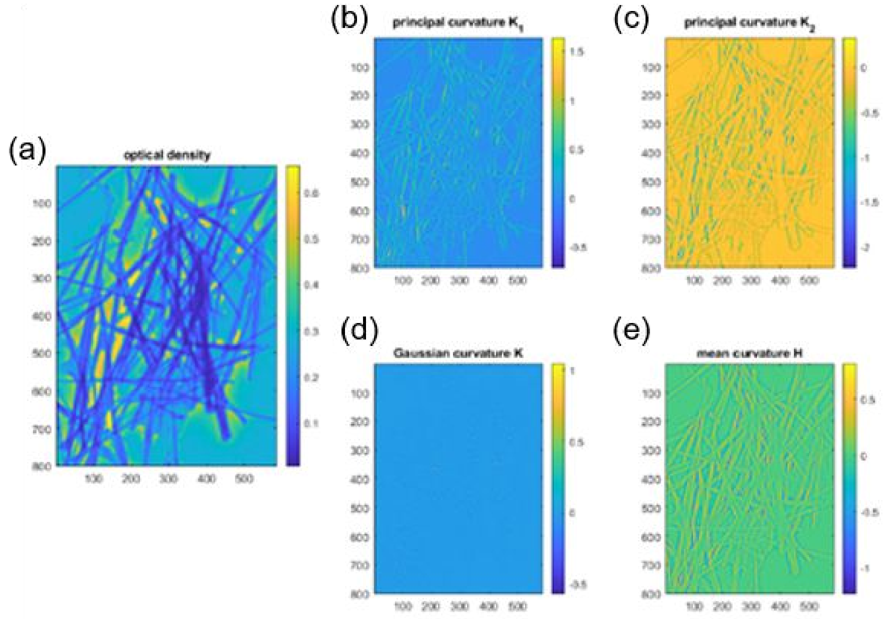

Figure S4: (a) Estimation of relative sample thickness from Beer's law smoothed with a Gaussian kernel. Image representation of (b) principal curvature K_1, (c) second principal curvature K_2, (d) Gaussian curvature $K = K_1 * K_2$, and (e) mean curvature $(K_1 + K_2)/2$.

Two characteristic quantities can be derived from those principal curvatures, the Gaussian curvature, which is the square of the geometric mean of K_1 and K_2,

$$K = K_1 K_2$$

and the mean curvature, which is the arithmetic mean of those

$$H = \frac{K_1+K_2}{2}.$$

Common examples are a saddle shaped surface, which has a negative Gaussian curvature, the surface of a top half of a hemisphere, which has positive Gaussian curvature, and the surface of a cylinder which has a finite mean curvature, but zero Gaussian curvature.

To take this mathematical approach we apply the following assumptions:

1. A uniform coating of the NWs
2. A uniform absorption of the NW giving a height according to Beer's law $I = I_o e^{-\mu d}$

Defining the height as a function of x and y coordinate, $z = d(x,y)$, and assuming smoothness meets the definition of a differentiable manifold and allows for quantitative calculation of curvature components (Fig. S4a). With these assumptions, we calculate principal curvatures K_1, K_2, Gaussian curvature K, and mean curvature H (Fig. S4 b-e). $K = \frac{LN-M^2}{EG-F^2}$ and $H = \frac{LG-2MF+NE}{2(EG-F^2)}$ are used to calculate mean and Gaussian curvature from the first and second fundamental form where $E = I(u,u), F = I(v,v), G = I(v,v), L = II(u,u), M = II(u,v), N = II(v,v)$. The surface is smoothed using a Gaussian kernel to prevent local noise artifacts (roughness) from dominating the curvature calculation.

SI 6. DW on the curved region

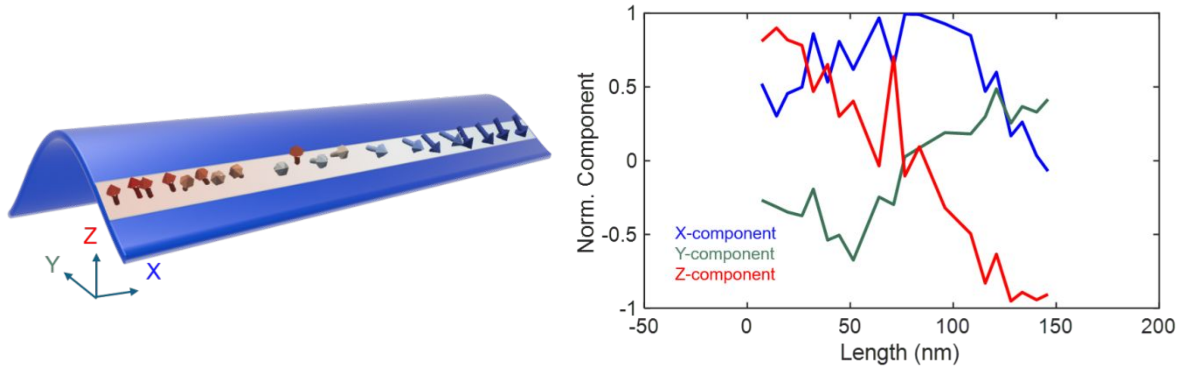

Figure S5: Domain wall and magnetization components at the right side of the curved film.

DW configuration and magnetization components at the top and one side (left) of the curved surface is shown in Fig. 4 of the main text. Fig. S5 shows DW and magnetization components on the opposite side (right) of the curved surface. Here, the *y*-component is opposite to that in Fig. 4. Note that, one DW is plotted in Fig. S5 while panels in Fig. 4 of the main text contain two DWs. This is because a majority of the domains end on the right side of the curved region.

References:

1. Sorrentino, A.; Nicolas, J.; Valcarcel, R.; Chichon, F. J.; Rosanes, M.; Avila, J.; Tkachuk, A.; Irwin, J.; Ferrer, S.; Pereiro, E., MISTRAL: a transmission soft X-ray microscopy beamline for cryo nano-tomography of biological samples and magnetic domains imaging. Journal of Synchrotron Radiation 2015, 22 (4), 1112-1117.
